# Accidental Cushing Syndrome

**DOI:** 10.1210/jcemcr/luad160

**Published:** 2024-01-02

**Authors:** Konstantina Kousoula, Oskar Ragnarsson, Penelope Trimpou

**Affiliations:** Department of Endocrinology, Sahlgrenska University Hospital, SE-41345 Gothenburg, Sweden; Department of Endocrinology, Sahlgrenska University Hospital, SE-41345 Gothenburg, Sweden; Department of Internal Medicine and Clinical Nutrition, Institute of Medicine at Sahlgrenska Academy, University of Gothenburg, SE-41345 Gothenburg, Sweden; Wallenberg Center for Molecular and Translational Medicine, University of Gothenburg, SE-41345 Gothenburg, Sweden; Department of Endocrinology, Sahlgrenska University Hospital, SE-41345 Gothenburg, Sweden; Department of Internal Medicine and Clinical Nutrition, Institute of Medicine at Sahlgrenska Academy, University of Gothenburg, SE-41345 Gothenburg, Sweden

**Keywords:** Cushing syndrome, accidental intake, exogenous Cushing syndrome, differential diagnosis

## Abstract

We present a patient with Cushing syndrome secondary to accidental intake of corticosteroid tablets—a 66-year-old woman with a history of well-controlled hypertension, who over the course of a few weeks developed full-blown Cushing syndrome with uncontrolled blood pressure, typical central fat accumulation, and easy bruising. The clinical features further worsened upon increase of the dosage of her antihypertensive medication because of rising blood pressure. Biochemical analyses showed low cortisol and ACTH concentrations. Inspection of the patient's medications revealed that she had accidentally been taking corticosteroids tablets, prescribed for her husband, instead of antihypertensives, ie, dexamethasone 4 mg and then 8 mg, instead of candesartan at the same dose.

This case highlights the necessity of a thorough review of the medications taken by patients suspected to have exogenous Cushing syndrome, including inspection of the original packaging, and not just relying on information from the patient and electronic health records. This case also highlights the need of special labeling on the packaging for the easy identification of corticosteroid-containing medications given their widespread availability.

## Introduction

Cushing syndrome (CS) is a disorder caused by prolonged and excessive exposure to glucocorticoids. The most common cause of CS is exogenous or iatrogenic, ie, CS caused by administration of glucocorticoids due to inflammatory, autoimmune, or neoplastic diseases. Endogenous CS is a rare condition, caused by either hypersecretion of ACTH from the pituitary gland, ectopic ACTH production, or hypersecretion of cortisol from the adrenal glands.

It is of great importance to exclude exogenous CS in all patients who present with signs and symptoms compatible with the syndrome. The following case highlights the need to rule out exogenous CS via a face-to-face review of the medications taken by a patient with CS, rather than only relying on the patient's history and electronic health record.

## Case Presentation

A 66-year-old woman was referred to our department for investigation of suspected CS. She was diagnosed with essential hypertension a couple of years earlier and was prescribed tablet candesartan 4 mg daily. Apart from an otherwise well-controlled hypertension, the patient had a history of bilateral hip replacement, the first performed in 2020 and the second 2 years later.

During the 6 weeks prior to our evaluation, the patient had noticed an increasing fat accumulation around her abdomen, upper back, neck, and over the collar bones, despite minimal increase of her body weight. Moreover, the patient had developed a rounded face and increased growth of facial hair, especially on the chin, as well as thin and fragile skin that bruised easily. About 1.5 weeks before she was referred to our clinic, the dose of candesartan was increased by her general practitioner from 4 to 8 mg daily because of rapidly worsening hypertension, confirmed by monitoring 24-hour ambulatory blood pressure.

## Diagnostic Assessment

**Figure 1.**
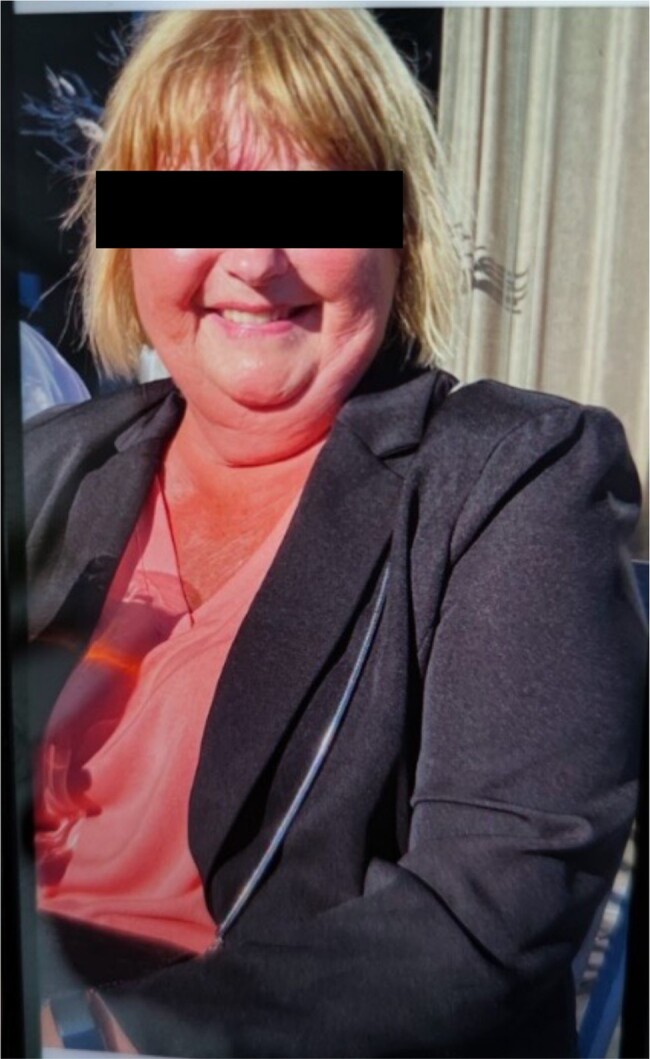
The patient few weeks prior to admission for evaluation of Cushing syndrome.

**Figure 2.**
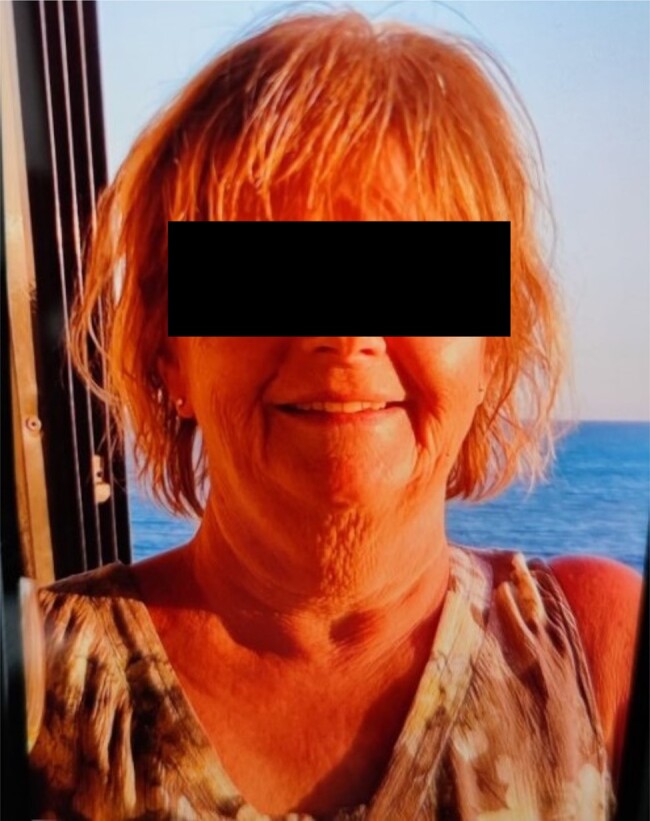
The patient many months before the onset of Cushing syndrome.

The physical examination of the patient revealed central obesity and multiple bruises that the patient could not recall. Increased growth of fine hairs on the chin and facial plethora was present. Blood pressure was 165/88 mmHg. The patient did not have any signs of abdominal stretch marks, nor did she have any obvious muscle wasting in the arms and legs (Fig. 1). When comparing to photographs taken about 6 months prior to the examination, the differences were obvious (Fig. 2).

Biochemical evaluation revealed unmeasurable plasma cortisol at 12:00 Pm, 4:00 Pm, and 6:00 Am (<28 nmol/L, reference 102-535 nmol/L; <1.01 μg/dL, reference 3.69-19.39 μg/dL). Serum ACTH was also undetectable (<0.2 pmol/L, reference 1.6-13.9 pmol/L; <0.91 pg/mL, reference 2.27-63.18 pg/mL), which raised suspicion of exogenous CS. The patient firmly denied any intake of anything other than her candesartan tablets. She even stated that she avoided any analgesics after the hip replacement previously the same year, nor had she received any intra-articular cortisone injection. The patient gave a very trustworthy and consistent impression, which inevitably led us to proceed to further investigation of the adrenal glands and the pituitary gland to exclude rarer forms of CS, such as cyclic CS and/or pituitary apoplexy of an ACTH-producing pituitary adenoma. The magnetic resonance imaging of the pituitary and the computed tomography of the adrenal glands were normal. Except for the low cortisol and ACTH levels, endocrine workup was unremarkable (Table 1).

**Table 1.** Biochemical evaluation of the patient with Cushing syndrome at baseline, ie, at admission

Hormone tested	Value	Normal Range
Plasma cortisol at 08:00 AM	**<1.01 mcg/dL (<28 nmol/L)**	3.70-19.39 mcg/dL (102-535 nmol/L)
ACTH	**<0.91 pg/mL (<0.2 pmol/L)**	7.27-63.18 pg/mL (1.6-13.9 pmol/L)
TSH	1.0 mIU/L (1.0 mIU/L)	0.4-3.7 mIU/L (0.4-3.7 mIU/L)
Free T4	1.01 ng/dL (13 pmol/L)	0.76-1.32 ng/dL (9.8-17 pmol/L)
IGF-1	142 ng/mL (18.60 nmol/L)	38-162 ng/mL (4.98-21.22 nmol/L)
Prolactin	374 mIU/L (17.58 mcg/L)	63-561 mIU/L (2.96-26.37 mcg/L)
FSH	90 mIU/mL (90 IU/L)	27-133 mIU/mL (post-menopausal) (27-133 IU/L)
LH	16 mIU/mL (16 IU/L)	5.2-62 mIU/mL (post-menopausal) (5.2-62 IU/L)
SHBG	6.07 mcg/mL (54 nmol/L)	2.25-17.42 mcg/mL (20-155 nmol/L)
Testosterone	**8.65 ng/dL (0.30 nmol/L)**	11.53-34.58 ng/dL (0.4-1.2 nmol/L)
Estradiol	<19.07 pg/mL (<70 pmol/L)	<28.06 pg/mL (<103 pmol/L) (post-menopausal with no hormone substitute)
Aldosterone	9.05 ng/dL 0.251 pmol/L	<23.61 ng/dL (recumbent position) <655 nmol/L
Renin	8.25 mIU/L	2.8-40 mIU/L (recumbent position)
DHEAS	**14.81 mcg/dL (0.4 µmol/L)**	29.63-181.48 mcg/dL (0.8-4.9 µmol/L)
HbA1c	45 mmol/mol (6.3 %)	31-46 mmol/mol (5-6.4 %)

Abnormal values are shown in bold font. Values in parenthesis are International System of Units (SI).

Abbreviations: ACTH, adrenocorticotropic hormone; TSH, thyroid-stimulating hormone; T4, thyroxine; IGF-1, insulin-like growth factor 1; FSH, follicle-stimulating hormone; LH, luteinizing hormone; SHBG, sex hormone binding globulin; DHEAS, dehydroepiandrosterone sulfate; HbA1c, glycated hemoglobin.

**Figure 3.**
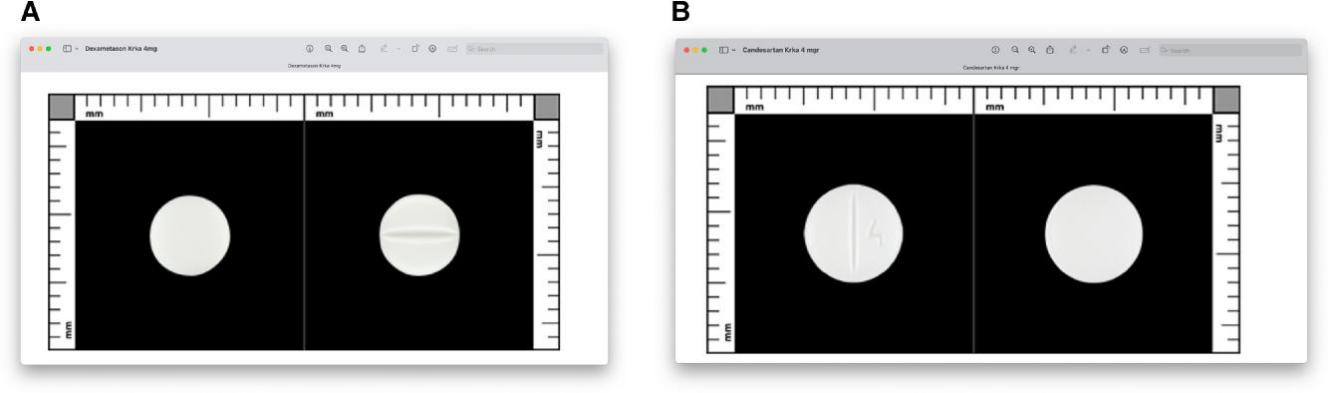
A. Tablet Dexamethasone 4 mg. White, scored, diameter 6 × 6 mm. B. Tablet Candesartan 4 mg. White, scored, diameter 7 × 7 mm.

On day 3 after admission, we noted that plasma cortisol at 8:00 Am was measurable, though still low, at 134 nmol/L (4.86 μg/dL), which reinforced our first suspicion of exogenous CS and prompted a more thorough review of the patient's medication. At this time, we asked the patient to show us the tablets that she had been taking at home and that she still carried in her purse. To the patient's frank surprise, it turned out that she was indeed carrying tablets containing 4 mg dexamethasone in the belief that they were candesartan 4 mg tablets. The dexamethasone 4 mg tablet the patient had (generic) was white, scored with a diameter of 6 mm (Fig. 3A). The candesartan 4 mg tablet the patient had been dispensed (generic) was also white, scored and with a diameter of 7 mm (Fig. 3B).

## Treatment

**Figure 4.**
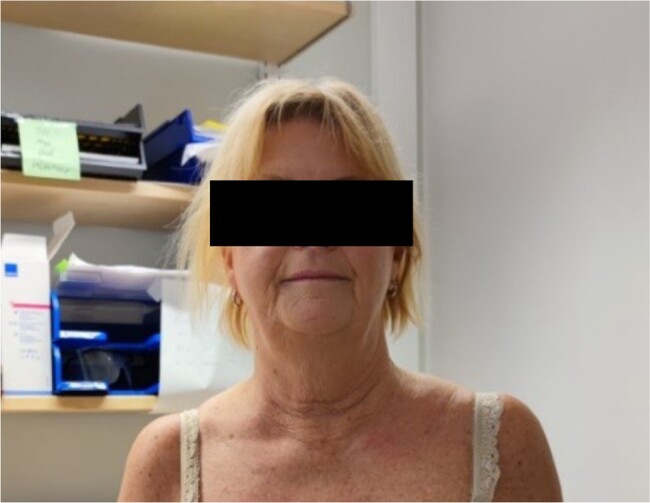
The patient 5 months after the resolution of Cushing syndrome.

The patient was discharged with the same antihypertensive medications as prior to the deterioration and referred to her general practitioner for follow-up of blood pressure. Upon clinical evaluation 5 months after discharge, she showed no signs or symptoms of CS (Fig. 4).

## Outcome and Follow-up

Thus, the patient had accidentally been taking her husband's medication, with which the patient had been aiding her husband, and developed a surreptitious iatrogenic CS. In hindsight, the severity of the clinical features had been worsening and resulted in rapid deterioration alongside the increase of the dosage of the antihypertensives from 4 to 8 mg because of the rising blood pressure.

By day 5 after admission, the patient's plasma cortisol and ACTH concentrations had normalized, as had her blood pressure.

## Discussion

Exogenous hypercortisolism is the most common cause of CS, though seldomly published in the literature, and is mainly iatrogenic because of prolonged use of high doses of synthetic glucocorticoids prescribed for the treatment of nonendocrine diseases (1). A recent study has shown that as many as every seventh resident in western Sweden received a glucocorticoid prescription between 2007 and 2014 (2).

The rising use of generic medications during the past decade has resulted in corticosteroids being available in different forms, shapes, and packages that make them less easily recognizable. In many countries, corticosteroids are available over-the-counter in almost any form, whereas a variety of agents such as herbal preparations, tonics, and skin-bleaching creams may also contain corticosteroids to the unawareness of the people using them (3, 4).

There are no large studies regarding how common the unintentional use of medicines or products that contain corticosteroids. However, studies on traditional Chinese medicine have shown that illegally impure herbs and medicines containing corticosteroids are widely used, suggesting that the accidental intake of corticosteroids is more frequent than we may think (3, 5). Many cases of factitious CS have been reported as a cause of exogenous CS, which makes the diagnosis even more challenging (6-8).

The Endocrine Society Clinical Practice Guidelines for the diagnosis of CS recommend that exogenous CS be always excluded before starting the investigation of endogenous CS (9). However, a specific and definitive approach for diagnosing, respectively excluding, exogenous CS is currently lacking. In a recent review, the authors recommend that in addition to asking the patient which medicines they take, the physician should review the electronic health record and ask particularly for medications that are administered via nonoral routes, as well as over-the-counter agents as mentioned earlier (10).

If not confirmed by history, the physician is advised to proceed to the measurement of ACTH and/or dehydroepiandrosterone sulfate as well as screening for synthetic glucocorticoids (10). The results usually show low ACTH, dehydroepiandrosterone sulfate, and cortisol levels even though the clinical picture suggests CS. The cross-reactivity of hydrocortisone or cortisone, which is similar to endogenous steroids, in immunoassay-based measurements of plasma and urinary cortisol may show variable levels of cortisol. These measurements combined with low ACTH can make the diagnostic workup much more complex (7). Screening for exogenous substances with the help of high-performance liquid chromatography is usually positive and constructive (7).

It is increasingly clear that the risk of accidental ingestion of potent medicines can have deleterious effects on health. This leads us to conclude that thorough face-to-face review of the packaging of medications taken by the patient is mandatory and can spare both physicians and patients from a series of unnecessary investigations. Given the high availability, easy access, and catastrophic adverse effects of the unintentional use of corticosteroids, we therefore propose that all corticosteroid-including medications and agents be marked with a recognizable label.

## Learning Points

- Exogenous CS should be always excluded before starting investigation of endogenous CS.
- Concerning exogenous CS, practitioners should always think broadly and ask for use of herbal preparations, skin-bleaching creams, and any over-the-counter products.
- Unintentional use of corticosteroids can still be the case even after a thorough review of the electronic records; practitioners should always inspect the medicines the patient has taken.

## Data Availability

Data sharing is not applicable to this article as no datasets were generated or analyzed during the current study.
